# Antibacterial Effect of Colloidal Suspensions Varying in Silver Nanoparticles and Ions Concentrations

**DOI:** 10.3390/nano12010031

**Published:** 2021-12-23

**Authors:** Varvara Platania, Alexandra Kaldeli-Kerou, Theodora Karamanidou, Maria Kouki, Alexander Tsouknidas, Maria Chatzinikolaidou

**Affiliations:** 1 Department of Materials Science and Technology, University of Crete, 70013 Heraklion, Greece; plataniavarvara@yahoo.com; 2 PLiN Nanotechnology S.A., Spectra Business Center 12th km Thessaloniki-Chalkidiki, Thermi, 57001 Thessaloniki, Greece; ak@plin-nanotechnology.com (A.K.-K.); tk@plin-nanotechnology.com (T.K.); vet@nanosanitas.com (M.K.); 3 Department of Mechanical Engineering, University of Western Macedonia, 50100 Kozani, Greece; 4 Foundation for Research and Technology Hellas (FO.R.T.H), Institute of Electronic Structure and Laser (IESL), 70013 Heraklion, Greece

**Keywords:** minimum inhibitory concentration, minimum bactericidal concentration, growth kinetics, *Escherichia coli*, *Pseudomonas aeruginosa*, *Staphylococcus aureus*, *Staphylococcus epidermidis*, nanosilver, microbiology

## Abstract

A lot of effort has been dedicated recently to provide a better insight into the mechanism of the antibacterial activity of silver nanoparticles (AgNPs) colloidal suspensions and their released silver ionic counterparts. However, there is no consistency regarding whether the antibacterial effect displayed at cellular level originates from the AgNPs or their ionic constitutes. To address this issue, three colloidal suspensions exhibiting different ratios of AgNPs/silver ions were synthesized by a wet chemistry method in conjunction with tangential flow filtration, and were characterized and evaluated for their antimicrobial properties against two gram-negative, *Escherichia coli* (*E. coli*) and *Pseudomonas aeruginosa* (*P. aeruginosa*), and two gram-positive, *Staphylococcus aureus* (*S. aureus*) and *Staphylococcus epidermidis* (*S. epidermidis*), bacterial strains. The produced samples contained 25% AgNPs and 75% Ag ions (AgNP_25), 50% AgNPs and 50% Ag ions (AgNP_50), and 100% AgNPs (AgNP_100). The sample AgNP_100 demonstrated the lowest minimum inhibitory concentration values ranging from 4.6 to 15.6 ppm for all four bacterial strains, while all three samples indicated minimum bactericidal concentration (MBC) values ranging from 16.6 ppm to 62.5 ppm against all strains. An increase in silver ions content results in higher bactericidal activity. All three samples were found to lead to a significant morphological damage by disruption of the bacterial cell membranes as analyzed by means of scanning electron microscopy (SEM). The growth kinetics demonstrated that all three samples were able to reduce the bacterial population at a concentration of 3.1 ppm. SEM and growth kinetic data underline that *S. epidermidis* is the most sensitive among all strains against the investigated samples. Our results showed that all three AgNPs colloidal suspensions exhibited strong antibacterial properties and, thus, they can be applied in medical devices and antimicrobial control systems.

## 1. Introduction

In recent years, a significant increase in the number of bacterial strains resistant to commercial antibiotics has been observed. Nowadays, over 70% of bacterial infections are caused by strains resistant to at least one type of the most commonly used drugs [1]. Numerous studies have evaluated the biocidal capacity of colloidal silver (Ag), advocating its effectiveness against both gram-negative and gram-positive bacteria [2], while suggested it as a valid alternative to conventional antiviral [3] and antiprotozoal agents [4]. Colloidal silver inherently refers to silver nanoparticles (AgNPs) suspended within a liquid; however, their diffusion and sedimentation results in ion formation [5], which reduces the mass of the particles in favor of their ionic counterparts. Consequently, AgNPs coexist with their silver ions, and silver content is conventionally defined by both the nanoparticles and the ions, rarely providing insight to their exact percentile allocation. This complicates the comparative evaluation of literature reports on the effectiveness of nanosilver due to an apparent lack of consensus as to whether this originates from AgNPs [6] or their ionic constitutes [7].

Four primary bactericidal mechanisms of colloidal silver are highlighted in the literature [8,9] including (i) bacteriolysis, triggering the destruction or dissolution of cellular membranes, (ii) generation of reactive oxygen species (ROS) and free radicals, (iii) destabilization of intracellular structures (e.g., mitochondria or ribosomes) and biomolecules (e.g., DNA and proteins), and (iv) modulation of signal transduction pathways. These mechanisms could be attributed to either AgNPs or cationic silver, despite their different chemical and physical structure [10,11,12].

The effectiveness of silver ions and AgNPs against gram-positive and gram-negative bacterial cells has been recently reviewed [13], suggesting different modes of action. Gram-negative bacteria were found to be more prone to silver ions [14], whereas initial evidence would suggest that AgNPs display a superior penetration ability into gram-positive bacteria [12]. However, size, shape [15], coating [16], and even production route [17] of the nanoparticles have been suggested to affect their biocidal capacity and, thus, a direct comparison of individual studies might be subject to methodological details. Another recent report on nanosilver coatings produced by flame aerosol direct deposition revealed that the silver ion concentration in solution mostly drives the antibiofilm activity [18], illustrating at the same time the size effect of the AgNPs on their antimicrobial effect, with the smallest ones around 6 nm and demonstrating the highest inhibition action against *S. aureus*.

Despite the effort dedicated to this subject [19], the current literature does not provide adequate insight into the activity displayed at a cellular level and whether this originates from the AgNPs or their ionic counterparts. To address this issue, antibacterial properties of three colloidal suspensions exhibiting different ratios of AgNPs/silver ions were evaluated in two gram-negative bacterial strains, *Escherichia coli* (*E. coli*) and *Pseudomonas aeruginosa* (*P. aeruginosa*), and two gram-positive strains, *Staphylococcus aureus* (*S. aureus*) and *Staphylococcus epidermidis* (*S. epidermidis*). The determination of the minimum inhibitory and minimum bactericidal concentration, as well as the bacterial morphology by means of scanning electron microscopy (SEM) were conducted, while the growth kinetics of four bacterial strains in the presence of different concentrations of the three agents were analyzed, aiming at providing a better insight into the varying antibacterial mechanisms exhibited at molecular level.

## 2. Materials and Methods

### 2.1. Nanoparticles Production

Silver nitrate (99.9% AgΝO_3_, Mr = 169.873 g/mol) was used as silver precursor and purchased from Duchefa Biochemie (Haarlem, The Netherlands). The reduction agent was produced by components conventionally found in the literature [20,21,22]. Two reagents were used for the stabilization of the nanoparticles: a protein with a molecular mass between 20,000 and 25,000 g/mol (Sigma Aldrich) and a non-ionic surfactant with a molecular mass between 1000 and 2000 g/mol (of a 98%–99% purity), both purchased from Alfa Aesar (Haverhill, MA, USA). All reagents were used as received without any further purification. Silver nanoparticles were synthesized through a wet chemistry approach by dissolving silver salt and reduction agents separately in deionized water. The silver salt solution was magnetically stirred along with the protein-based stabilizer up to a predefined temperature to ensure complete dissolution. The reduction agent was then added to the aqueous solution of the silver precursor whilst stirring. The color of the solution changed to dark orange, thus indicating the formation of AgNPs, and the non-ionic surfactant was added to the colloid.

### 2.2. Quantification of Silver Content

The as-produced colloidal suspension had a silver content of 1500 ppm. Tangential Flow Filtration (TFF) was employed to increase nanoparticle concentration up to 5 times. During this process, the colloid flows tubularly past a membrane, which the silver-ions permeate along with other organic constituents (smaller than the membrane’s pore size), while the nanoparticles are retained within the circular flow [23]. Towards this, a membrane pore size of 5 kDa (Pall Corporation) was rinsed through hot sanitization with sodium hydroxide (NaOH, 0.1–0.25 M). The filter was flushed for each sample set with deionized water for 25 min to ensure full removal of the NaOH prior to filtration of the AgNPs. Following this, the silver content of the supernatant solution (e.g., high in silver ion content) and that of the retentate containing the AgNPs were evaluated through Inductively Coupled Plasma—Optical Emission Spectrometry (ICP-OES). Three different samples were prepared, containing 25% (AgNP_25), 50% (AgNP_50), and 100% (AgNP_100) silver nanoparticles, respectively.

### 2.3. Physicochemical Characterization

The physicochemical characteristics of the produced AgNPs suspensions were assessed in terms of particle size and distribution profiles, obtained by Dynamic Light Scattering (DLS) using a VASCO 3 DLS analyzer (Cordouan Technologies, Pessac, France). The UV-Vis spectra of the samples were determined, upon dilution, using a Cary 60 UV-Vis (Agilent Technologies, Santa Clara, CA, USA). 

### 2.4. Preparation of Bacterial Suspension

The bacterial strains of two gram-negative *E. coli* and *P. aeruginosa* and two gram-positive *S. epidermidis* and *S. aureus* bacteria were inoculated and cultivated in the CLSI standard Mueller–Hinton Broth (MH-B) and incubated at 37 °C for 18–20 h at 200 rpm. The concentration of bacterial suspension was then determined by the optical density at 600 nm using a UV-Vis spectrophotometer and diluted for further tests. All bacterial strains were maintained as glycerol stocks at −80 °C.

### 2.5. Minimal Inhibitory Concentration (MIC) and Minimal Bactericidal Concentration (MBC)

MIC and MBC were determined using a broth microdilution method according to the Clinical and Laboratory Standards Institute (CLSI) protocol, as previously described [24]. Briefly, bacteria were streaked from frozen glycerol stocks onto non selective LB agar plates and incubated overnight at 37 °C. Colonies from the fresh plates were suspended in MH broth and grown at 37 °C in a shaking incubator at 200 rpm. Before each experiment, the optical density of the cell suspension was normalized to the optical density OD600 of 0.1, which corresponds to 2 × 10^7^ colony forming units (CFU) per mL through serial dilutions [25]. The bacterial cell number was then adjusted in the broth to a concentration of 2 × 10^6^ CFU/mL, 50 μL of which was added to triplicate wells of a 96-well plate, containing 50 μL of AgNPs to various concentrations from a stock of 200 ppm. The final concentrations tested were 100, 50, 25, 12.5, 6.25, 3.125, 1.562, 0.781, and 0.39 ppm. The plates were incubated overnight at 37 °C. The MIC values present the lowest concentrations at which each of the triplicate wells (*n* = 3) in each 96-well plate was clear after 16–24 h of incubation. The MBC was determined by plating, on LB agar plates, the wells with the concentrations that indicated growth inhibition (clear), and noting the lowest concentration that resulted in lack of growth after an overnight incubation at 37 °C. The results are presented as mean of triplicates of three independent experiments (*n* = 9).

### 2.6. Tolerance Level

The tolerance levels of each bacterial strain against AgNPs were determined according to the method of May et al. [26] using the following formula:

$$Tolerance=\frac{MBC}{MIC}$$

### 2.7. Morphological Observation

The morphology of *E. coli*, *P. aeruginosa*, *S. epidermidis*, and *S. aureus* cultured with the material suspensions for 3, 6, and 24 h, was observed by Scanning Electron Microscope (SEM). The bacterial suspension contained (1–2) × 10^8^ CFU/mL and was prepared as previous described [27]. Briefly, stocks of the materials diluted in MH-B were prepared at a concentration of 200 ppm and were added to bacterial suspension until the final concentration was 100 ppm. The samples were incubated at 37 °C and after 3, 6, and 24 h, 100 μL aliquot were centrifuged and the pellet washed with PBS. Every sample was then fixed with 100 μL of 4% *v*/*v* para-formaldehyde on nitrocellulose (NC) filter membranes with a 0.2 μm pore size for 15 min and then dehydrated in increasing concentrations (30–100% *v*/*v*) of ethanol. Samples were finally dried using hexamethyl disilazane (HMDS), sputter-coated with a 20 nm thick layer of gold (Baltec SCD 050), and observed under a scanning electron microscope at an accelerating voltage of 15 kV (JEOL JSM-6390 LV, Peabody, MA, USA).

### 2.8. Kinetic Study

For the kinetic study, 50 μL of bacterial suspension at a concentration of 2 × 10^5^ CFU/mL were placed in each well of a 96-well plate. Stock solutions of AgNPs diluted in MH-B were prepared at a concentration of 100 ppm. The final concentrations tested were 50, 25, 12.5, 6.25, and 3.125 ppm. The bacterial suspension in pure MH-B and in the presence of amoxicillin/clavulanic acid (50 ppm) was taken as negative and positive control respectively. For the growth kinetic experiments of *P. aeruginosa*, streptomycin (100 ppm) was used as positive control due to resistance in amoxicillin/clavulanic acid. The 96-well plate was transferred to a spectrophotometer (Synergy HTX Multi-Mode Microplate Reader) and the absorbance was measured at 600 nm every 15 min for 24 h. All experiments were performed in triplicate. The results are presented as mean of triplicates of three independent experiments (*n* = 9).

### 2.9. Statistical Analysis

All experiments were performed in triplicate. The results are presented as mean of triplicates of three independent experiments (*n* = 9). Statistical analysis and graph plotting was conducted using GraphPad Prism 8.

## 3. Results

### 3.1. Physicochemical Characterization

**Figure 1:**
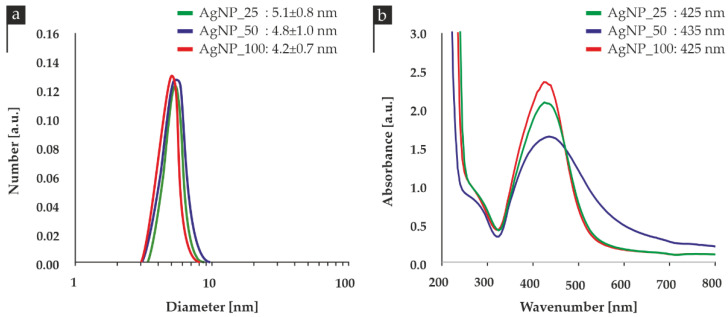
(**a**) Particle size distribution and (**b**) UV-Vis spectra of all three AgNPs samples.

The size distribution of the nanoparticles in all three samples is demonstrated in Figure 1a, indicating the presence of a similar-sized and monodisperse populations of nanoparticles. UV-Vis spectroscopy revealed absorption peaks at wavelengths between 425–435 nm, as illustrated in Figure 1b.

Table 1 provides an overview of the quantification of the three tested samples in terms of AgNPs and their solvated ions.

The physicochemical characteristics of all three colloids are summarized in Table 2. The antibacterial activity of AgNPs depends primarily on size, ionic strength of the medium, and the type of capping agent. According to the literature, differences in antibacterial action are presented between silver nanoparticles synthesized in acidic and basic medium. However, these results are mostly related to the size of AgNPs, which is affected by the pH of the medium during the synthesis process. Our samples presented an acidic pH and similar diameters and were evaluated for the effect of different ion/particle ratio with respect to their antibacterial activity.

### 3.2. Minimal Inhibitory Concentration (MIC) and Minimal Bactericidal Concentration (MBC) and Tolerance Level

The antibacterial action of the three colloidal AgNPs suspensions against both gram-negative (*E. coli*, *P. aeruginosa*) and gram-positive (*S. aureus, S. epidermidis*) microorganisms at different concentrations revealed a strong dose-dependent antimicrobial activity against all four strains (Table 3).

The antimicrobial activity (MIC and MBC) of AgNPs was absent up to 4.6 ppm against all of the bacterial strains. The MIC values of AgNPs were in the range of 4.6 to 20.8 ppm, and MBC values for all three samples ranged from 16.6 to 100.0 ppm. The AgNP_100 sample demonstrated the lowest MIC values in three out of four tested bacteria, indicating a greater inhibition effect compared with AgNP_25 and AgNP_50. To avoid possible misinterpretations due to the turbidity of insoluble compounds into the broth dilution tube, MBC was determined by culturing the MIC dilutions on the sterile LB agar plates. The MBC values against all bacteria range from 16.6 ppm to 56.2 ppm, except of *S. aureus* with MBC values of 100.0 ppm (AgNP_50) and 62.5 ppm (AgNP_100). *S. epidermidis* expressed the lowest MBC value against AgNP_50 sample at 16.6 ppm, followed by both AgNP_100 and AgNP_25 at 25 ppm. *E. coli* also presented an early bactericidal effect for AgNP_25 treatments at 25 ppm.

The tolerance level of each strain against various concentrations of AgNPs was calculated from the respective MIC and MBC values (Table 4). In general, the tolerance levels against AgNP_50 sample were the lowest. The implication is that bactericidal agents kill microbes, whereas bacteriostatic agents simply inhibit the bacterial growth. When the MBC/MIC ratio is greater than or equal to 16 for bacteria, the antimicrobial agent is considered bacteriostatic and when this ratio is less than or equal to four, then the agent is considered bactericidal [28]. The National Clinical Committee for Laboratory Standards (NCCLS) further suggests that an agent is bactericidal when it causes greater than a 3-log (99.9%) reduction in colony-forming units (CFU)/mL after 18–24 h of incubation in liquid media. MBC is usually identical to or within one or two doubling dilutions of the MIC; if the MBC exceeds the MIC by 32-fold or more, the microbe is defined as tolerant. The MBC/MIC ratio is a parameter that reflects the bactericidal capacity of the analyzed compound. In our study, tolerance values ranged from 1.6 to 8.0, implicating strong antibacterial properties against all four tested bacteria. Treatment with AgNP_25 exerted a bactericidal effect for all strains, and AgNP_50 proved to be bactericidal for all strains except of the gram-positive *S. aureus*. AgNP_100 demonstrated a bactericidal effect only for gram-negative bacteria *E. coli* and *P. aeruginosa.*

### 3.3. Morphological Observation of E. coli, P. aeruginosa, S. aureus and S. epidermidis

**Figure 2:**
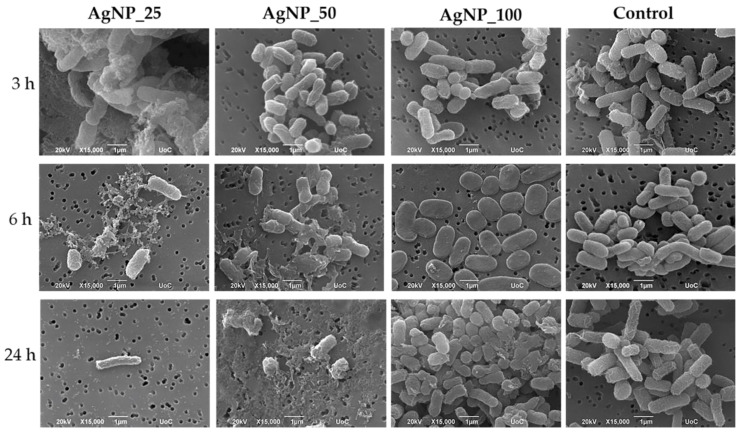
Representative SEM images showing the induction of 100 ppm AgNPs on *E. coli* for 3, 6, and 24 h. The control represents the morphology of the bacteria without AgNPs. Magnification is 15,000× and scale bar represents 1 μm.

**Figure 3:**
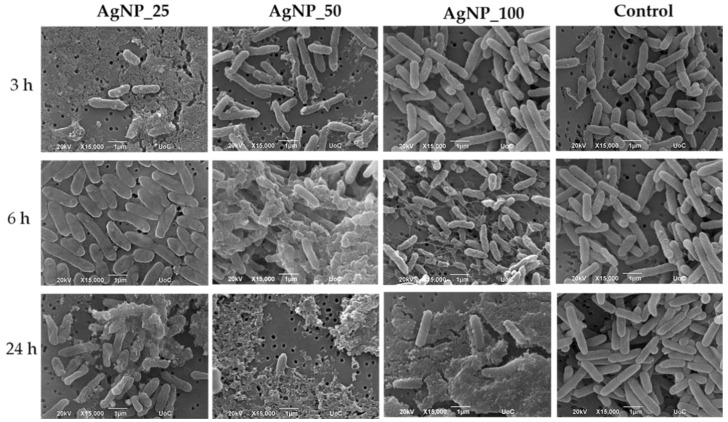
Representative SEM images showing the induction of 100 ppm AgNPs on *P. aeruginosa* for 3, 6 and 24 h. The control represents the morphology of the bacteria without AgNPs. Magnification is 15,000× and scale bar represents 1 μm.

**Figure 4:**
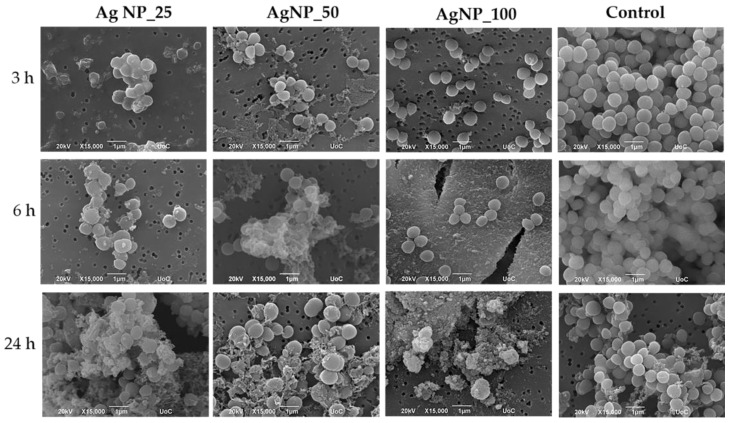
Representative SEM images showing the induction of 100 ppm AgNPs on *S. aureus* for 3, 6, and 24 h. The control represents the morphology of the bacteria without AgNPs. Magnification is 15,000× and scale bar represents 1 μm.

**Figure 5:**
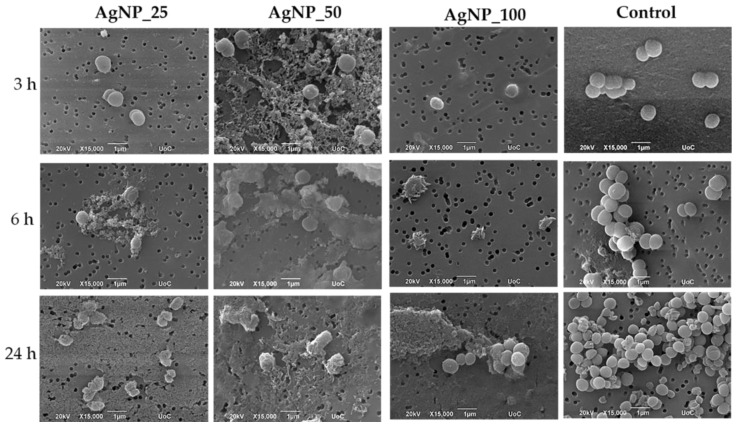
Representative SEM images showing the induction of 100 ppm AgNPs on *S. epidermidis* for 3, 6, and 24 h. The control represents the morphology of the bacteria without AgNPs. Magnification is 15,000× and scale bar represents 1 μm.

The morphological changes of gram-negative *E. coli* and *P. aeruginosa* and gram-positive *S. aureus* and *S. epidermidis* bacterial cell membranes under the induction of 100 ppm AgNPs samples for 3, 6, and 24 h were observed under SEM and are shown in Figure 2, Figure 3, Figure 4 and Figure 5. The morphological changes were compared to non-treated bacteria. Micrographs by SEM depict that the surface of bacterial cells of untreated control group was smooth and showed typical characteristics of the surface of native cells, while cells treated with AgNPs appeared severely damaged. Some cells showed large leakage, and other appear misshapen and fragmentary.

As shown in Figure 2, after 3, 6, and 24 h of incubation, *E. coli* bacterial cells appeared with a flattened morphology with damaged cell membranes when treated with all three AgNPs samples, in contrast to the physiological rod-shaped control. For AgNP_25 and AgNP_50, a decrease in cell number corresponding to the incubation time was observed. Overall, an increase of silver ions content leads to more extensive morphological damage and decrease of cell number.

*P. aeruginosa* presented a flattened morphology compared with the untreated rod-shaped control bacteria. After 24 h of incubation, the shrinkage of the bacterial membrane was dramatically increased (Figure 3). *P. aeruginosa* cells in direct contact with samples AgNP_25 and AgNP_50 were severely affected even after short incubation periods of 3 and 6 h compared with AgNP_100-treated cells.

SEM images for *S. aureus* and *S. epidermidis* confirmed that the surface of bacterial cells of the untreated control group exhibited characteristics typical to those of native cells and appeared smooth and intact, while cells treated with AgNPs were damaged severely. Some cells showed large leakage, and other appear misshapen and fragmentary.

Even more pronounced was the effect on the cell membranes of *S. epidermidis,* in which an extensive cell membrane disruption, fragmented morphology, and agglomerate formation were observed. Induction of all AgNP samples caused a dramatic decrease in bacterial cell number for the first 3 h.

### 3.4. Bacterial Growth Kinetics at Various AgNPs Concentrations

**Figure 6:**
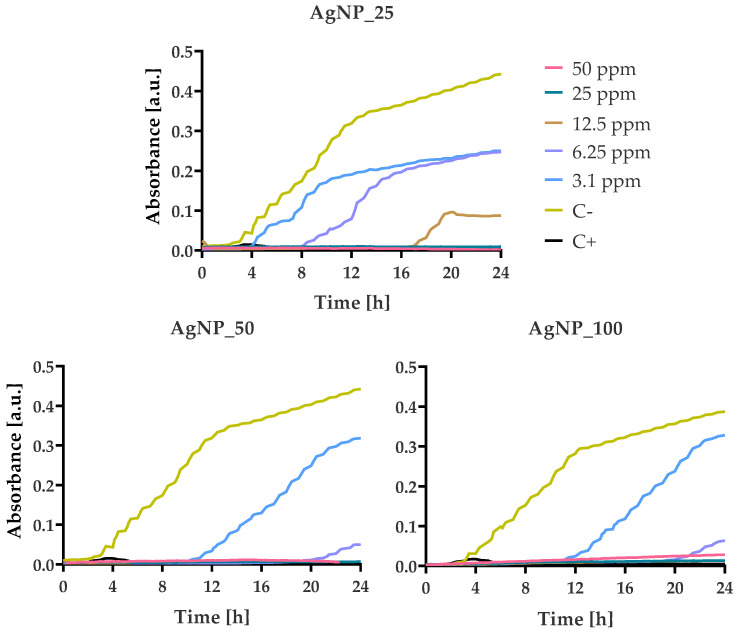
Growth kinetics of *E. coli* in the presence of different concentrations (from 3.125 to 50 ppm) of AgNPs. Optical density was measured for 24 h at 37 °C using a multi-detection microplate reader at 600 nm and automatically recorded for each well every 15 min. In all experiments, the bacterial suspension in pure MH-B and in the presence of amoxicillin/clavulanic acid (50 ppm) were taken as negative and positive control, respectively.

**Figure 7:**
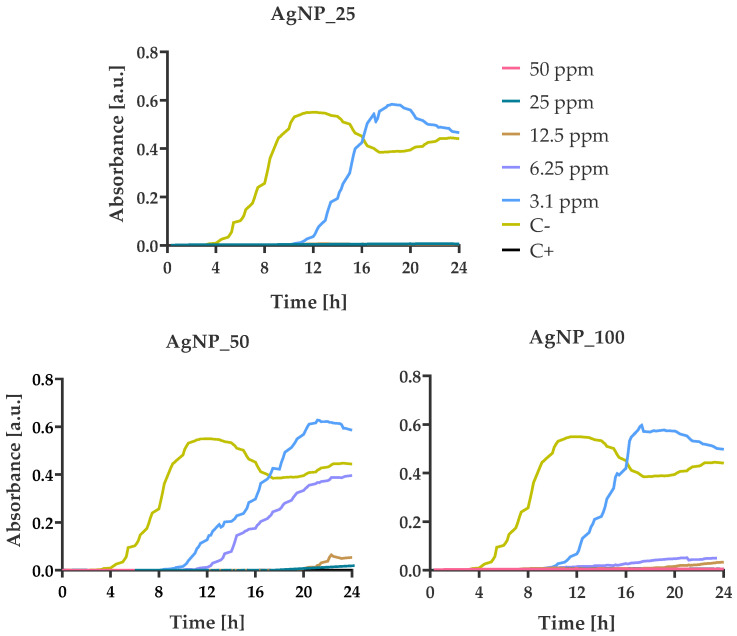
Growth kinetics of *P. aeruginosa* in the presence of different concentrations (from 3.125 to 50 ppm) of AgNPs. Optical density was measured for 24 h at 37 °C using a multi-detection microplate reader at 600 nm and automatically recorded for each well every 15 min. In all experiments, the bacterial suspension in pure MH-B and in the presence of streptomycin (100 ppm) were taken as negative and positive control, respectively.

**Figure 8:**
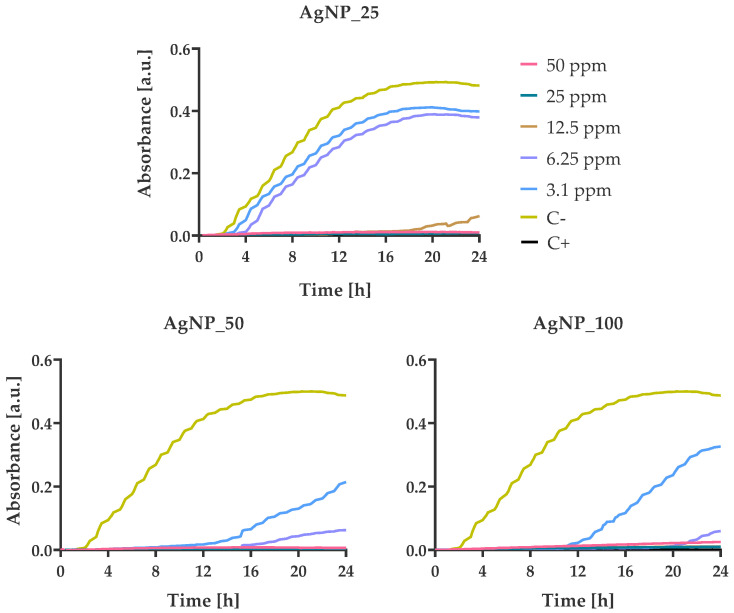
Growth kinetics of *S. aureus* in the presence of different concentrations (from 3.125 to 50 ppm) of AgNPs. Optical density was measured for 24 h at 37 °C using a multi-detection microplate reader at 600 nm and automatically recorded for each well every 15 min. In all experiments, the bacterial suspension in pure MH-B and in the presence of amoxicillin/clavulanic acid (50 ppm) were taken as negative and positive control respectively.

**Figure 9:**
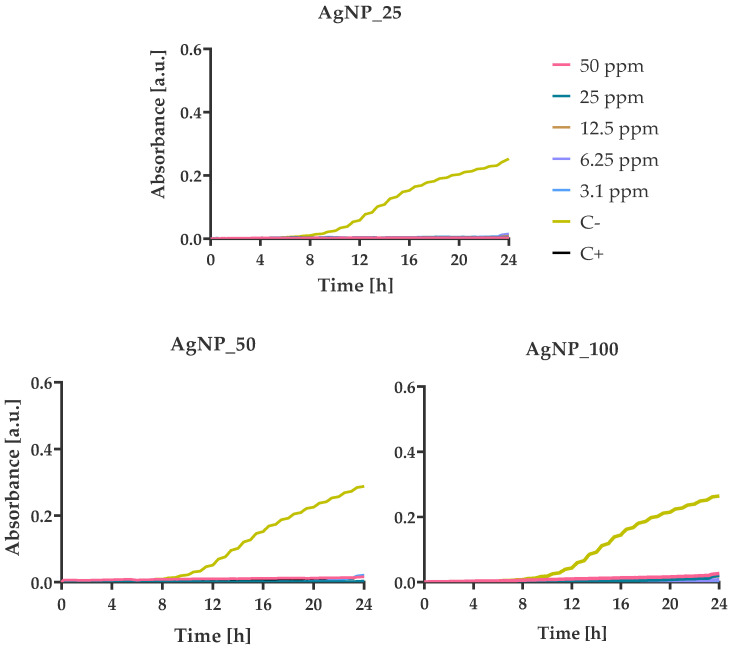
Growth kinetics of *S. epidermidis* in the presence of different concentrations (from 3.125 to 50 ppm) of AgNPs. Optical density was measured for 24 h at 37 °C using a multi-detection microplate reader at 600 nm and automatically recorded for each well every 15 min. In all experiments, the bacterial suspension in pure MH-B and in the presence of amoxicillin/clavulanic acid (50 ppm) were the negative and positive control, respectively.

The growth kinetics of *E. coli*, *P. aeruginosa*, *S. aureus*, and *S. epidermidis* were monitored in 100 μL MH-B supplemented with final concentrations of 50, 25, 12.5, 6.25, and 3.125 ppm AgNPs, which were incubated for 24 h (Figure 6, Figure 7, Figure 8 and Figure 9).

Absorbance values at 600 nm reflect the number of bacterial cells. The values were found to increase in a sigmoidal fashion in the absence of AgNPs. For *E. coli* cells, the usual sigmoidal growth was observed at 0 ppm concentration of AgNPs (C-). The presence of low AgNPs concentrations (3.125, 6.25, and 12.5 ppm) resulted in partial inhibition of the growth of the *E. coli* cells, as demonstrated by the extended lag phases (Figure 6). The increase of AgNPs percentage seemed to increase the delay of the bacterial growth from 2 h (AgNP_25) to 8 h (AgNP_50) and 10 h (AgNP_100), causing a profound bactericidal effect from 12.5 ppm (AgNP_25) to 6.25 ppm (AgNP_50) and 3.1 ppm (AgNP_100) for the first 24 h.

For the growth kinetic experiments of *P. aeruginosa*, streptomycin (100 μg/mL) was used as positive control due to its resistance in amoxicillin/clavulanic acid. For all treatments, *P. aeruginosa* cells presented a growth kinetics delay of 6 h in the presence of 3.1 ppm of AgNPs samples in comparison with the untreated control C- (Figure 7). After 12 h of incubation, there was an increase in growth at 6.25 ppm of the AgNP_50 sample.

The growth kinetics of *S. aureus* demonstrated a similar trend to *E. coli* for all tested AgNPs samples (Figure 8). Based on the kinetic diagrams, similarities were observed in the extended lag phases with a delay from 1 h (AgNP_25) to 8 h (AgNP_50) and 10 h (AgNP_100) and a robust bactericidal effect from 12.5 ppm (AgNP_25) to 6.25 ppm (AgNP_50) and 3.1 ppm (AgNP_100).

*S. epidermidis* is the strain that presented the highest sensitivity of all four tested bacteria against all three AgNPs samples. The incubation of *S. epidermidis* with all tested concentrations led to a bactericidal effect against *S. epidermidis* cells during the first 24 h (Figure 9). These results are in good agreement with the morphological observation by SEM images showing the complete damage of the bacterial cell membranes (Figure 5).

## 4. Discussion

The AgNPs have been reported to exhibit a broad spectrum of antibacterial activities on both gram positive and gram-negative bacteria and various drug-resistant strains. The release of silver ion has been reported to be higher when fine AgNPs with a particle size of less than 10 nm are used for antibacterial action compared to larger AgNPs [29]. As dissolution of silver ions directly released from the AgNPs into the samples may affect their biocidal profile, the determination of the colloidal silver content is of significant importance; thus, it was the subject of this study. Although the exact mechanism of the antibacterial effect of AgNPs has not been entirely clarified, various antibacterial actions have been proposed. Among them, the most common modes of action can be attributed to (i) free silver ions uptake causing interruption of ATP molecules and preventing DNA replication, or (ii) formation of reactive oxygen species by AgNPs, or (iii) direct damage of cell membrane by silver ions [8,24]. It has been generally recognized that AgNPs form holes in the bacterial cell wall, causing increased permeability and cell death. Since AgNPs cause denaturation and oxidize the cell wall, they lead to rupture of organelles, resulting in cell lysis [30]. The tangential flow filtration (TFF) employed in this study to increase the concentration of nanoparticles up to five times was considered as the preferable procedure for the separation process, as conventional methods such as ultracentrifugation or dialysis tubes may favor the ionic generation, or provide artificially high ionic concentrations, and thus lead to allocation inconsistencies [31]. Following the filtration of the AgNPs by TFF, the content of the supernatant containing the silver ions and that of the retentate containing the AgNPs were evaluated through ICP-OES, leading to the production of three colloidal solutions comprising 25% AgNPs and 75% Ag ions (AgNP_25), 50% AgNPs and 50% Ag ions (AgNP_50), and 100% AgNPs (AgNP_100). The antibacterial properties of these three colloidal solutions containing different ratios of AgNPs and silver ions were evaluated against two gram-negative, *E. coli* and *P. aeruginosa* and two gram-positive, *S. aureus* and *S. epidermidis* bacterial strains, in an attempt to elucidate whether variations in silver nanoparticle and silver ion concentrations have an effect on the antimicrobial action.

The results presented here are partially in agreement with the literature, as Ag ions had higher toxicity to *E. coli* than AgNPs [32]. Although other studies have suggested that silver ions have a similar, yet more pronounced mode of action than the AgNPs [33], this was not a trend that could be verified in all evaluation methods performed in this study. AgNPs exhibited a lower minimal inhibitory concentration against gram-positive bacteria than colloids with increased ionic silver, which is in alignment with the literature, suggesting that silver ions are more effective against gram-negative bacterial cells [15]. In particular, the sample AgNP_100 demonstrated with 4.6 to 15.6 ppm the lowest MIC values for all four strains, which may suggest a greater inhibition effect in general for increased Ag nanoparticle concentrations. Looking into the results of the sample AgNP_100 on the MBC, we observed similar values ranging from 16.6 ppm to 56.2 ppm against both gram-negative strains and the gram-positive strain *S. epidermidis*, implicating its strong antibacterial activity. The *S. aureus* is an exception for which the MBC values were found to be higher, namely 62.5 ppm for AgNP_100, and 100.0 ppm for AgNP_50. These results could be interpreted due to the thick peptidoglycan layer of the gram-positive bacterium *S. aureus*. In addition, our data on the tolerance, calculated as the MBC/MIC ratio, range from 1.6 to 8.0, implicating strong antibacterial properties against all four tested bacteria. Specifically, gram-positive bacteria depict a greater tolerance to all three colloidal samples compared to the gram-negative strains. Moreover, tolerance values indicated that treatment with AgNP_25 exerted a bactericidal effect for all four bacterial strains, while AgNP_50 was found to be bactericidal for all strains except of the gram-positive *S. aureus*, and AgNP_100 demonstrated a bactericidal effect only against the gram-negative bacteria *E. coli* and *P. aeruginosa*, underlying that an increase in silver ion content leads to a higher bactericidal activity against gram-positive bacteria.

The growth kinetics of *E. coli, P. aeruginosa* and *S. aureus* incubated with the three samples demonstrated a similar trend. All three colloids, even at the lower concentrations of 3.1 ppm, were able to reduce the final bacterial population. Based on the growth kinetic diagrams, an increased nanoparticle concentration seems to be related to a better inhibition effect of the bacterial growth. Lower concentrations of AgNP_100 (<3.1 ppm) and AgNP_50 (<6.25 ppm) inhibited the growth of bacterial strains for more than 24 h compared with AgNP_25.

Comparing all four bacterial strains employed in this study, *S. epidermidis* demonstrated a great sensitivity to all three AgNP samples. *S. epidermidis* presented the lowest MBC value at 16.6 ppm in the presence of AgNP_50, followed by a value at 25 ppm under the induction of both samples, the AgNP_100 and the AgNP_25. An extensive bacterial membrane damage and dramatic decrease of cell number from the first 3 h of incubation were observed by means of SEM. The incubation with all tested concentrations led to a bactericidal effect against *S. epidermidis* cells during the first 24 h as evidenced from the kinetic studies. All experimental results indicated that *S. epidermidis* is more affected by the action of the three agents than the other three investigated bacterial strains, which may be attributed to the particle size of 5 nm of the AgNPs produced in this work. The highest cytotoxic activity against *S. epidermidis* has been reported by other research groups as being caused by AgNPs with a particle size below 10 nm [34]. Previous work on *S. epidermidis* suggested that exposure to silver ions promotes the formation of the lethal hydroxyl radical in a process believed to result from the inhibition of electron transport chain components and production of the superoxide anion [35], justifying the extensive cell membranes damage observed in our case. Overall, it can be stated that the particle size of less than 10 nm enabling higher permeability through the cell membrane, together with the formation of reactive hydroxyl species from the silver ions, may have led to the highest antibacterial action of the colloidal agents against *S. epidermidis*. In addition, our results indicated a strong antibacterial activity of all three formulations against *P. aeruginosa*, with a comparable effect on cell membrane damage. Based on literature reports, *P. aeruginosa* displays resistance to a variety of antibiotics, including aminoglycosides, quinolones, and β-lactams [36]. Generally, the major mechanisms of *P. aeruginosa* used to counter antibiotic attack can be classified into intrinsic, acquired, and adaptive resistance [37,38]. Moreover, multidrug-tolerant cells that are able to survive antibiotic attack can form biofilms; these cells are responsible for prolonged and recurrent infections [39]. The development of new antibiotics or alternative therapeutic strategies for the treatment of *P. aeruginosa* infections is urgently required for the patients with resistant infections to conventional antibiotics. New antibiotics with novel modes of action have been explored in recent years, as they have new routes of administration and resistance to modification by bacterial enzymes. AgNPs are considered potential agents to help manage and prevent infections [27] towards this direction, and this is in line with our data.

## 5. Conclusions

The antibacterial effect of Ag ions was distinguished from that of AgNPs by monitoring the growth of two gram-negative and two gram-positive bacterial strains in the presence of three different samples, containing different ratios of AgNPs and Ag ions. The prepared samples comprised 25% AgNPs and 75% Ag ions (AgNP_25), 50% AgNPs and 50% Ag ions (AgNP_50), and 100% AgNPs (AgNP_100). The particle size was determined to be approximately 5 nm for all three sample compositions. We observed that both AgNPs and Ag ions displayed a strong antibacterial activity. An increase of the AgNPs concentration resulted in a stronger inhibitory effect of the bacterial growth, while an increase in Ag ions content displayed higher bactericidal properties. In our study, gram-positive *S. epidermidis* expressed great sensitivity to all tested samples. These results suggest that the produced colloidal suspensions comprising AgNPs and Ag ions can be used as efficient antibacterial agents in biomedical applications.

## Figures and Tables

**Table 1:** Silver content allocation.

Sample	% Ag NPs	% Ag ions
AgNP_25	~25%	~75%
AgNP_50	~50%	~50%
AgNP_100	100%	-

**Table 2:** Physicochemical characterization of AgNPs.

Sample	Size (nm)	pH
AgNP_25	5.1 ± 0.8	3.92
AgNP_50	4.8 ± 1.0	3.55
AgNP_100	4.2 ± 0.7	3.91

**Table 3:** MIC and MBC values of AgNPs solutions for *E. coli, P. aeruginosa, S. epidermidis*, *and S. aureus* expressed in ppm.

	*E. coli*		*P. aeruginosa*		*S. aureus*		*S. epidermidis*	
	MIC	MBC	MIC	MBC	MIC	MBC	MIC	MBC
AgNP_25	12.3	25.0	12.5	50.0	12.3	50.0	12.5	25.0
AgNP_50	20.8	50.0	16.6	41.6	12.5	100.0	10.4	16.6
AgNP_100	15.6	56.2	12.5	31.3	9.4	62.5	4.6	25.0

**Table 4:** Tolerance values of *E. coli, P. aeruginosa, S. epidermidis*, and *S. aureus* against AgNPs samples.

	AgNP_25	AgNP_50	AgNP_100
*E. coli*	2.0	2.4	3.6
*P. aeruginosa*	4.0	2.5	2.5
*S. aureus*	4.0	8.0	6.7
*S. epidermidis*	2.0	1.6	5.3

## Data Availability

Data supporting reported results will be provided by the authors upon request.

## References

[1] Dizaj S.M., Lotfipour F., Barzegar-Jalali M., Zarrintan M.H., Adibkia K. (2014). Antimicrobial activity of the metals and metal oxide nanoparticles. Mater. Sci. Eng. C Mater. Biol. Appl..

[2] Vila Domínguez A., Ayerbe Algaba R., Miró Canturri A., Rodríguez Villodres Á., Smani Y. (2020). Antibacterial Activity of Colloidal Silver against Gram-Negative and Gram-Positive Bacteria. Antibiotics.

[3] Galdiero S., Falanga A., Vitiello M., Cantisani M., Marra V., Galdiero M. (2011). Silver nanoparticles as potential antiviral agents. Molecules.

[4] Adeyemi O.S., Molefe N.I., Awakan O.J., Nwonuma C.O., Alejolowo O.O., Olaolu T., Maimako R.F., Suganuma K., Han Y., Kato K. (2018). Metal nanoparticles restrict the growth of protozoan parasites. Artif. Cells Nanomed. Biotechnol..

[5] Teeguarden J.G., Hinderliter P.M., Orr G., Thrall B.D., Pounds J.G. (2007). Particokinetics in vitro: Dosimetry considerations for in vitro nanoparticle toxicity assessments. Toxicol. Sci..

[6] Dasgupta N., Ramalingam C. (2016). Silver nanoparticle antimicrobial activity explained by membrane rupture and reactive oxygen generation. Environ. Chem. Lett..

[7] Park H.J., Kim J.Y., Kim J., Lee J.H., Hahn J.S., Gu M.B., Yoon J. (2009). Silver-ion-mediated reactive oxygen species generation affecting bactericidal activity. Water Res..

[8] Marambio-Jones C., Hoek E.M.V. (2010). A review of the antibacterial effects of silver nanomaterials and potential implications for human health and the environment. J. Nanoparticle Res..

[9] Dakal T.C., Kumar A., Majumdar R.S., Yadav V. (2016). Mechanistic Basis of Antimicrobial Actions of Silver Nanoparticles. Front. Microbiol..

[10] Chen R., Ni H., Zhang H., Yue G., Zhan W., Xiong P. (2013). A preliminary study on antibacterial mechanisms of silver ions implanted stainless steel. Vacuum.

[11] Morones J.R., Elechiguerra J.L., Camacho A., Holt K., Kouri J.B., Ramírez J.T., Yacaman M.J. (2005). The bactericidal effect of silver nanoparticles. Nanotechnology.

[12] Lok C.N., Ho C.M., Chen R., He Q.Y., Yu W.Y., Sun H., Tam P.K., Chiu J.F., Che C.M. (2006). Proteomic analysis of the mode of antibacterial action of silver nanoparticles. J. Proteome Res..

[13] Kędziora A., Speruda M., Krzyżewska E., Rybka J., Łukowiak A., Bugla-Płoskońska G. (2018). Similarities and Differences between Silver Ions and Silver in Nanoforms as Antibacterial Agents. Int. J. Mol. Sci..

[14] Sütterlin S., Tano E., Bergsten A., Tallberg A.B., Melhus A. (2012). Effects of silver-based wound dressings on the bacterial flora in chronic leg ulcers and its susceptibility in vitro to silver. Acta Derm.-Venereol..

[15] Pal S., Tak Y.K., Song J.M. (2007). Does the antibacterial activity of silver nanoparticles depend on the shape of the nanoparticle? A study of the Gram-negative bacterium *Escherichia coli*. Appl. Environ. Microbiol..

[16] Coleman J.G., Kennedy A.J., Bednar A.J., Ranville J.F., Laird J.G., Harmon A.R., Hayes C.A., Gray E.P., Higgins C.P., Lotufo G. (2013). Comparing the effects of nanosilver size and coating variations on bioavailability, internalization, and elimination, using Lumbriculus variegatus. Environ. Toxicol. Chem..

[17] Ge L., Li Q., Wang M., Ouyang J., Li X., Xing M.M.Q. (2014). Nanosilver particles in medical applications: Synthesis, performance, and toxicity. Int. J. Nanomed..

[18] Geissel F.J., Platania V., Gogos A., Herrmann I.K., Belibasakis G.N., Chatzinikolaidou M., Sotiriou G.A. (2022). Antibiofilm activity of nanosilver coatings against Staphylococcus aureus. J. Colloid Interface Sci..

[19] Shang L., Dong S., Nienhaus G.U. (2011). Ultra-small fluorescent metal nanoclusters: Synthesis and biological applications. Nano Today.

[20] Van Dong P., Ha C.H., Binh L.T., Kasbohm J. (2012). Chemical synthesis and antibacterial activity of novel-shaped silver nanoparticles. Int. Nano Lett..

[21] Chekin F., Ghasemi S. (2014). Silver nanoparticles prepared in presence of ascorbic acid and gelatin, and their electrocatalytic application. Bull. Mater. Sci..

[22] Christy C., Adams G., Kuriyel R., Bolton G., Seilly A. (2002). High-performance tangential flow filtration: A highly selective membrane separation process. Desalination.

[23] Maurer E.I., Sharma M., Schlager J.J., Hussain S.M. (2014). Systematic analysis of silver nanoparticle ionic dissolution by tangential flow filtration: Toxicological implications. Nanotoxicology.

[24] Manouras T., Platania V., Georgopoulou A., Chatzinikolaidou M., Vamvakaki M. (2021). Responsive Quaternized PDMAEMA Copolymers with Antimicrobial Action. Polymers.

[25] Sezonov G., Joseleau-Petit D., D’Ari R. (2007). Escherichia coli physiology in Luria-Bertani broth. J. Bacteriol..

[26] May J., Shannon K., King A., French G. (1998). Glycopeptide tolerance in Staphylococcus aureus. J. Antimicrob. Chemother..

[27] Skandalis N., Dimopoulou A., Georgopoulou A., Gallios N., Papadopoulos D., Tsipas D., Theologidis I., Michailidis N., Chatzinikolaidou M. (2017). The Effect of Silver Nanoparticles Size, Produced Using Plant Extract from Arbutus unedo, on Their Antibacterial Efficacy. Nanomaterials.

[28] Woods G., Washington J. (1995). The clinician and the microbiology laboratory. Principles and Practice of Infectious Diseases.

[29] Roco M.C. (2004). Nanoscale Science and Engineering: Unifying and Transforming Tools. AIChE J..

[30] Sondi I., Salopek-Sondi B. (2004). Silver nanoparticles as antimicrobial agent: A case study on *E. coli* as a model for Gram-negative bacteria. J. Colloid Interface Sci..

[31] Choi Y., Kim H.A., Kim K.W., Lee B.T. (2018). Comparative toxicity of silver nanoparticles and silver ions to Escherichia coli. J. Environ. Sci..

[32] Nägeli K.V. (1893). Über oligodynamische Erscheinungen in lebenden Zellen. Neue Denkschr. Der Allg. Schweiz. Ges. Für Die Gesamten Nat..

[33] Li W.-R., Sun T.-L., Zhou S.-L., Ma Y.-K., Shi Q.-S., Xie X.-B., Huang X.-M. (2017). A comparative analysis of antibacterial activity, dynamics, and effects of silver ions and silver nanoparticles against four bacterial strains. Int. Biodeterior. Biodegrad..

[34] Swolana D., Kępa M., Idzik D., Dziedzic A., Kabała-Dzik A., Wąsik T.J., Wojtyczka R.D. (2020). The Antibacterial Effect of Silver Nanoparticles on Staphylococcus epidermidis Strains with Different Biofilm-Forming Ability. Nanomaterials.

[35] Gordon O., Vig Slenters T., Brunetto P.S., Villaruz A.E., Sturdevant D.E., Otto M., Landmann R., Fromm K.M. (2010). Silver coordination polymers for prevention of implant infection: Thiol interaction, impact on respiratory chain enzymes, and hydroxyl radical induction. Antimicrob. Agents Chemother..

[36] Hancock R.E., Speert D.P. (2000). Antibiotic resistance in Pseudomonas aeruginosa: Mechanisms and impact on treatment. Drug Resist. Updates.

[37] Breidenstein E.B., de la Fuente-Núñez C., Hancock R.E. (2011). Pseudomonas aeruginosa: All roads lead to resistance. Trends Microbiol..

[38] Drenkard E. (2003). Antimicrobial resistance of Pseudomonas aeruginosa biofilms. Microbes Infect..

[39] Mulcahy L.R., Burns J.L., Lory S., Lewis K. (2010). Emergence of Pseudomonas aeruginosa strains producing high levels of persister cells in patients with cystic fibrosis. J. Bacteriol..

